# Small Molecule Treatments Improve Differentiation Potential of Human Amniotic Fluid Stem Cells

**DOI:** 10.3389/fbioe.2021.623886

**Published:** 2021-02-22

**Authors:** Aistė Zentelytė, Deimantė Žukauskaitė, Ieva Jacerytė, Veronika V. Borutinskaitė, Rūta Navakauskienė

**Affiliations:** Department of Molecular Cell Biology, Life Sciences Center, Institute of Biochemistry, Vilnius University, Vilnius, Lithuania

**Keywords:** amniotic fluid stem cells, neurogenic differentiation, small molecules, trichostatin A, vitamin C, retinoic acid

## Abstract

Human amniotic fluid stem cells (AFSC) are an exciting and very promising source of stem cells for therapeutic applications. In this study we investigated the effects of short-term treatments of small molecules to improve stem cell properties and differentiation capability. For this purpose, we used epigenetically active compounds, such as histone deacetylase inhibitors Trichostatin A (TSA) and sodium butyrate (NaBut), as well as multifunctional molecules of natural origin, such as retinoic acid (RA) and vitamin C (vitC). We observed that combinations of these compounds triggered upregulation of genes involved in pluripotency (*KLF4*, *OCT4*, *NOTCH1*, *SOX2*, *NANOG*, *LIN28a*, *CMYC*), but expression changes of these proteins were mild with only significant downregulation of Notch1. Also, some alterations in cell surface marker expression was established by flow cytometry with the most explicit changes in the expression of CD105 and CD117. Analysis of cellular energetics performed using Seahorse analyzer and assessment of gene expression related to cell metabolism and respiration (*NRF1*, *HIF1*α, *PPARGC1A*, *ERR*α, *PKM*, *PDK1*, *LDHA, NFKB1, NFKB2, RELA, RELB, REL*) revealed that small molecule treatments stimulate AFSCs toward a more energetically active phenotype. To induce cells to differentiate toward neurogenic lineage several different protocols including commercial supplements N2 and B27 together with RA were used and compared to the same differentiation protocols with the addition of a pre-induction step consisting of a combination of small molecules (vitC, TSA and RA). During differentiation the expression of several neural marker genes was analyzed (*Nestin*, *MAP2*, *TUBB3*, *ALDH1L1*, *GFAP, CACNA1D, KCNJ12, KCNJ2, KCNH2*) and the beneficial effect of small molecule treatment on differentiation potential was observed with upregulated gene expression. Differentiation was also confirmed by staining TUBB3, NCAM1, and Vimentin and assessed by secretion of BDNF. The results of this study provide valuable insights for the potential use of short-term small molecule treatments to improve stem cell characteristics and boost differentiation potential of AFSCs.

## Introduction

Nowadays, alternative sources of potent stem cells are of great interest and human amniotic fluid could be an attractive option. Stem cells isolated from amniotic fluid display several key characteristics that are essential for therapeutic applications. Amniotic fluid stem cells (AFSC) possess the ability to self-renew, differentiate into cell lineages from all three germ layers, they do not form teratomas *in vivo* and have low immunogenicity (Bossolasco et al., 2006; De Coppi et al., 2007; Roubelakis et al., 2007; Da Sacco et al., 2011). These cells are autogenous to the fetus and semiallogeneic to each parent and are said to be more potent than stem cells from other sources, such as bone marrow, umbilical cord blood, endometrium and other (Yan et al., 2013; Bonaventura et al., 2015; Alessio et al., 2018). Although AFSCs are somewhat similar to pluripotent stem cells, they are still considered as multipotent stem cells, and one approach to improve the plasticity and applicability of AFSCs could be by using small molecules. The use of small molecules is a relatively new technique of cell reprogramming and transdifferentiation (Kim et al., 2020). Nuclear transfer, transcription factor transfection, mRNA based reprogramming methods face many challenges in time and yield efficiency, complexity of delivery and the risk of integrating exogenous genetic material. Meanwhile, small molecules are inexpensive and easy to apply and control time, concentration and combination wise, they usually easy to manufacture and have a long shelf life. In addition, small molecules are cell permeable, non-immunogenic and can be prescribed to patients as drugs to promote endogenous cell repair and regeneration (Baranek et al., 2017; Ma et al., 2017). Our study was designed to determine how our selected epigenetically active compounds affect stem cell characteristics, such as surface marker and pluripotency associated gene expression, as well as what effect small molecules of interest have on metabolic phenotype and neurogenic differentiation of AFSCs. The aim of this study was to investigate whether uncomplicated and short-term treatments with small molecules improve stem cell characteristics and also provide an improved differentiation efficiency of AFSCs toward neurogenic lineage. We investigated the impact of the following small molecules on primary stem cell lines: HDAC inhibitors trichostatin A (TSA), sodium butyrate (NaBut) and multifunctional molecules of natural origin retinoic acid (RA) and vitamin C (vitC). We demonstrated that the concentrations and combinations of small molecules do not have a cytotoxic effect on AFSCs, but they do affect gene expression patterns with an increased expression of pluripotency markers and neurogenesis associated transcription factors (*OCT4*, *NANOG*, *LIN28a*, *CMYC*, *NOTCH1*, *SOX2*), although at protein level the changes are mild except for significant downregulation of Notch1. Also, small molecules affect the expression of surface markers (SSEA4, CD117, TRA-1-81, CD105). Treated stem cells with combinations of small molecules showed altered metabolic profile as evident by the changes in mitochondrial and glycolytic activity and expression of genes involved in cellular metabolism (*NRF1*, *HIF1*α, *PPARGC1A*, *ERR*α, *PKM*, *PDK1*, and *LDHA*) and NF-κB pathway (*NFKB1, NFKB2, RELA, RELB, REL*). To test the small molecule combination treatments, we examined the neurogenic differentiation potential of AFSCs and showed that adding a pre-induction step of small molecule treatment improved secretion levels of BDNF and the expression of tested neurogenic genes (*Nestin*, *MAP2*, *TUBB3*, *ALDH1L1*, *GFAP*) and genes of several ion channels (*CACNA1D, KCNJ12, KCNJ2, KCNH2*). In summary, this study demonstrates that short treatments with small molecule combinations could be used as pre-induction steps to improve differentiation efficiency.

## Materials and Methods

### Isolation and Expansion of Human Amniotic Fluid Stem Cells

Human amniotic fluid samples were obtained by amniocentesis from healthy women (age average—39 years) who needed prenatal diagnostics, but no abnormalities were detected by genetic and karyotype analysis (protocols approved by the Ethics Committee of Biomedical Research of Vilnius District, No 158200-123-428-122). AFSC were isolated using two-step isolation protocol as previously described (Tsai et al., 2004; Savickiene et al., 2015). Isolated cells were maintained in DMEM media, supplemented with 10% fetal bovine serum (FBS), 100 U/ml penicillin and 100 μg/ml streptomycin (Gibco, Thermo Fisher Scientific). To observe cells in culture, phase contrast microscope (Nicon Eclipse TS100) was used.

### Flow Cytometry Analysis

AFSCs were characterized by the expression of their surface markers. Cells were collected and washed twice with phosphate buffered saline (PBS) with 1% bovine serum albumin (BSA). A total of 6⋅10^4^ cells/sample were resuspended in 50 μl of PBS with 1% BSA and incubated with the following mouse anti-human antibodies: phycoerythrin (PE) conjugated CD166 (Biolegend) TRA-1-60 (Invitrogen), fluorescein isothiocyanate (FITC) conjugated CD34, CD73, CD90, CD105 (Biolegend), Alexa Fluor^®^ 488 conjugated CD31, HLA-ABC, HLA-DR (Biolegend), allophycocyanin (APC) conjugated CD44, CD117, CD146, SSEA4 (Biolegend), TRA-1-81 (Invitrogen). Appropriate mouse isotype controls were used, such as IgG1-PE (Biolegend), IgM-PE (Invitrogen), IgG1-FITC, IgG2a-FITC (Biolegend), IgG1-Alexa Fluor^®^ 488, IgG2a-Alexa Fluor^®^ 488 (Biolegend), IgG1-APC, IgG3-APC (Biolegend), IgM-APC (Invitrogen). Labeled cells were incubated in the dark at 4°C for 30 min, then washed twice with PBS with 1% BSA and then analyzed. For intracellular staining cells were washed with PBS and fixed using 2% paraformaldehyde at RT for 10 min. After washing step cells were permeabilized with 0.1% Triton X-100 in PBS/1% BSA solution at RT for 15 min. After centrifugation cells were resuspended in permeabilization solution and incubated for 30 min at 4°C in the dark with the following mouse anti-human antibodies: Alexa Fluor^®^ 488 conjugated Sox2 (Biolegend), Alexa Fluor^®^ 647 conjugated Nanog, Oct4 (Biolegend), unconjugated Lin28a and c-Myc. Goat anti-mouse IgG (H + L) Highly Cross-Absorbed Alexa Fluor^®^ 488 (Invitrogen) conjugated secondary antibodies were used to label Lin28a and c-Myc samples for another 30 min at 4°C in the dark. After incubations cells were washed with PBS/1% BSA and analyzed. The measurements were performed using BD FACSCanto^TM^ II flow cytometer with BD FACSDIVA^TM^ software (BD Biosciences). Ten thousand events were collected for each sample.

### Karyotyping AFSCs

To determine origin of AFSCs, karyotype analysis was performed and only samples with confirmed male fetus were chosen. AFSCs were treated with 0.6 μg/ml of colchicine for 3 h, then cells were collected by trypsinization and incubated with pre-warmed hypotonic 0.55% KCl solution for 30 min. at 37°C. The cells were fixed with fixation solution consisting of methanol and glacial acetic acid (3:1) at −20°C for 30 min., centrifuged and fixation repeated two more times. A few drops of cell suspension were transferred on a microscope slide and stained with 5% Giemsa (Merck) solution in Sorensen’s phosphate buffer for 5 min. Slides were analyzed at a magnification of 1,000× (Nikon ECLIPSE E200). Only well-spread metaphases with 42 ± 1 chromosomes were used for the analysis.

### Treatment With Agents and MTT Assay

Cells were seeded into 48-well plates and treated with different concentrations and combinations of epigenetically active compounds [Decitabine, Trichostatin A (TSA), Sodium butyrate (NaBut), Retinoic acid (RA) and Vitamin C (VitC)], three replicates each. After incubation periods the medium was removed from the cells and to measure cell viability 100 μl of 0.2 mg/ml MTT reagent in PBS (Sigma-Aldrich) was added to each well and then incubated for 1.5 h at 37°C. The precipitate was dissolved in 200 μl 96% ethanol and optical density (OD) of each well was measured using spectrophotometer Infinite M200 Pro (Tecan) at 570 and 630 nm wavelength. Cell viability (in percent) was calculated as the ratio between ODs (570 and 630 nm) of treated samples and untreated control.

### Neurogenic Differentiation

To differentiate AFSCs toward neurogenic lineage, several induction protocols were used. Induction medias consisted of DMEM/F12 with GlutaMax^TM^, 3 μM of RA (Sigma-Aldrich), 100 U/ml penicillin and 100 μg/ml streptomycin and either 1% of N2 supplement, 2% B27 supplement or their combination (Gibco, Thermo Fisher Scientific). Cells were induced to differentiate at 40–60% confluence with media changes every 3 days. To test the effect of epigenetically active molecules, a pre-treatment step was added. AFSCs were treated for 24 h with 25 μg/ml VitC, 20 nM TSA, 0.1 μM RA in DMEM/F12 supplemented with 5% FBS and 100 U/ml penicillin and 100 μg/ml streptomycin. Then the pretreatment media was changed to differentiation medias. I—1% of N2 and 3 μM of RA, II—2% B27 and 3 μM of RA, III—1% of N2, 2% B27 and 3 μM of RA, VI—preinduction, then 1% of N2 and 3 μM of RA, V—preinduction, then 2% B27 and 3 μM of RA, VI—preinduction, then 1% of N2, 2% B27 and 3 μM of RA. Morphological changes were observed with phase contrast microscopy. More information on optimization of neurogenic differentiation media and differentiation induction design is provided in Supplementary Figures 1, 2.

### Gene Expression Analysis

Total RNA from AFSCs was isolated using TRIzol^®^ reagent (Applied Biosystems) as recommended by the manufacturer. For the gene expression analysis, cDNA synthesis was performed using SensiFAST^TM^ cDNA Synthesis Kit (Bioline). RT-qPCR was performed with SensiFAST^TM^ SYBR^®^ No-ROX Kit (Bioline) on the Rotor-Gene 6000 thermocycler with Rotor-Gene 6000 series software (Corbett Life Science). *GAPDH* gene was used for normalization of the mRNA amount and the relative gene expression was calculated using ΔΔCt method (compared to untreated or undifferentiated control). The list of primers (Metabion International AG, Planegg-Steinkirchen, Germany) is provided in Supplementary Material.

### Extracellular Flux Analysis

Control and treated cells were characterized by their energetic profile which was determined using Seahorse XFp Extracellular Flux Analyzer and Cell Energy Phenotype Test Kit (Agilent Technologies, CA, United States). Oxygen consumption rate (OCR) and extracellular acidification rate (ECAR) were measured simultaneously firstly without inhibitors (the baseline), and then after the addition of oligomycin and FCCP (inhibitors of the electron transfer chain). After the measurements cell protein lysates were obtained using RIPA buffer (150 mM NaCl, 10 mM EDTA, pH 8.0, 10 mM Tris, pH 7.4, 0.1% SDS, 1% deoxycholate, 1% NP-40 in PBS, pH 7.6) and protein concentrations were measured with spectrophotometer Infinite M200 Pro (Tecan, Switzerland) using DC Protein Assay (Bio-Rad Laboratories, CA, United States). OCR and ECAR values were normalized to the total amount of protein in each well. Cell energy phenotype as the ratio of normalized OCR to normalized ECAR (OCR/ECAR) and the metabolic potential as the percentage increase of stressed OCR over baseline OCR and stressed ECAR over baseline ECAR, were assessed from Cell Energy Phenotype Test data using Seahorse Wave Desktop Software.

### Immunofluorescence Analysis

To assess neurogenic differentiation, AFSCs were seeded on coverslips and cultivated as undifferentiated control or differentiated toward neurogenic lineage using I-VI protocols for 14 days. Cells were with 4% paraformaldehyde for 15 min at RT and permeabilized using 0.2% Triton X-100 in PBS for 20 min. at RT. After washing with PBS, cells were blocked using 1% BSA/10% goat serum/PBS for 30 min at 37°C. Detection of NCAM: cells were incubated with primary mouse antibodies against NCAM1 (15 μg/ml) (Abcam) and secondary goat anti-mouse IgG (H + L) Highly Cross-Adsorbed, Alexa Fluor^®^ 594 antibodies (1:400) (Invitrogen) for 1 h each at 37°C in a humid chamber. Detection of TUBB3 and Vimentin: cells were incubated with FITC conjugated rabbit anti-beta III tubulin antibodies (1:100) (Abcam) or Alexa Fluor^®^ 488 conjugated rabbit anti-Vimentin antibodies (1:150) (Abcam) for 1 h at 37°C in a humid chamber. F-actin was labeled with Alexa Fluor^®^ 594 Phalloidin (Thermo Fisher Scientific) for 30 min RT. After each incubation coverslips were washed several times with PBS/1% BSA. Nuclei were stained using 300 nM DAPI solution (Invitrogen) for 10 min RT. Coverslips were mounted using Dako Fluorescent Mounting Medium (Agilent Technologies) and analyzed using Zeiss Axio Observer (Zeiss) fluorescent microscope, ×63 magnification with immersion oil and Zen BLUE software.

### Enzyme-Linked Immunosorbent Assay of BDNF

ELISA method was used to determine the secreted levels of BDNF in conditioned media of control AFSCs and AFSCs differentiated toward neurogenic lineage. Cells were seeded in wells of 6-well plates and were cultivated as control or induced to differentiate toward neurogenic lineage using I-VI protocols. Cells were washed with PBS and NutriStem^®^ hPSC XF medium (Biological Industries) was added for 3 days, after which both the cells and the media were collected separately. BDNF detection kit was purchased from R&D Systems and all procedures were carried out according to the manufacturer instructions. Plates were read with spectrophotometer Infinite M200 Pro (Tecan). AFSC protein lysates were obtained using RIPA buffer (150 mM NaCl, 10 mM EDTA, pH 8.0, 10 mM Tris, pH 7.4, 0.1% SDS, 1% deoxycholate, 1% NP-40 in PBS, pH 7.6) and protein concentrations were measured with Infinite M200 Pro using DC Protein Assay (BioRad Laboratories). BDNF values were normalized to total amount of cell protein.

### Statistical Analysis

All experiments were repeated at least 3 times (three independent experiments). Data were expressed as mean values with SDs. For statistical analysis, repeated measures analysis of variance with Tukey’s multiple comparison test or two-way analysis of variance with Bonferroni post-test in the GraphPad Prism Software (La Jolla, CA) was used.

## Results

### Characterization of Isolated AFSCs

**FIGURE 1:**
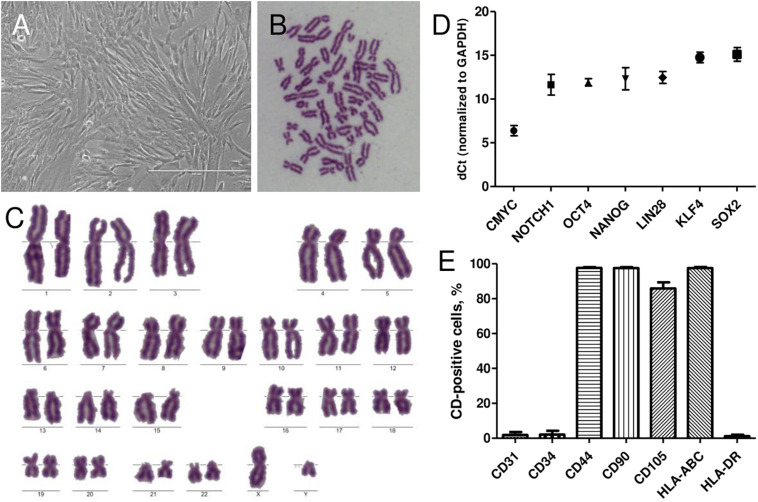
Characterization of isolated human AFSCs. **(A)** Isolated cells displayed the typical spindle-shaped morphology in monolayer culture. Scale bar 400 μm. **(B,C)** Metaphase spread and karyotype of AFSCs with male fetus. **(D)** Relative expression of stemness genes *CMYC*, *NOTCH1*, *OCT4*, *NANOG*, *LIN28a*, *KLF4*, and *SOX2* as determined by RT-qPCR. mRNA expression levels normalized to *GAPDH* are presented as mean ± SD (*n* = 3). **(E)** The expression of endothelial cells marker CD31, hematopoietic cells marker CD34, mesenchymal stem cells markers CD44, CD90, CD105, and immunological markers HLA-ABC, HLA-DR as measured by flow cytometry. Results are presented as mean ± SD (*n* = 3).

AFSCs were isolated from amniocentesis samples of healthy donors at midsecond (17–20 weeks) trimester of gestation. Stem cells were successfully isolated by a two-step protocol and when grown in culture demonstrated spindle-shaped morphology (Figure 1A). To confirm the fetal origin of the cells karyotype analysis was performed (Figures 1B,C). Only the samples with male fetus were chosen and Y chromosome was present in all instances. Relative expression of stemness markers *CMYC*, *NOTCH1*, *OCT4*, *NANOG*, *LIN28a*, *KLF4*, and *SOX2* was also detected by RT-qPCR (Figure 1D). AFSCs were strongly positive (over 90%) for mesenchymal cell surface markers, such as CD44, CD90, and CD105 and immunological marker HLA-ABC, negative (<5%) for hematopoietic marker CD34, endothelial marker CD31 and immunological marker HLA-DR (Figure 1E) as measured by flow cytometry.

### Evaluation of Small Molecule Effects on AFSCs

**FIGURE 2:**
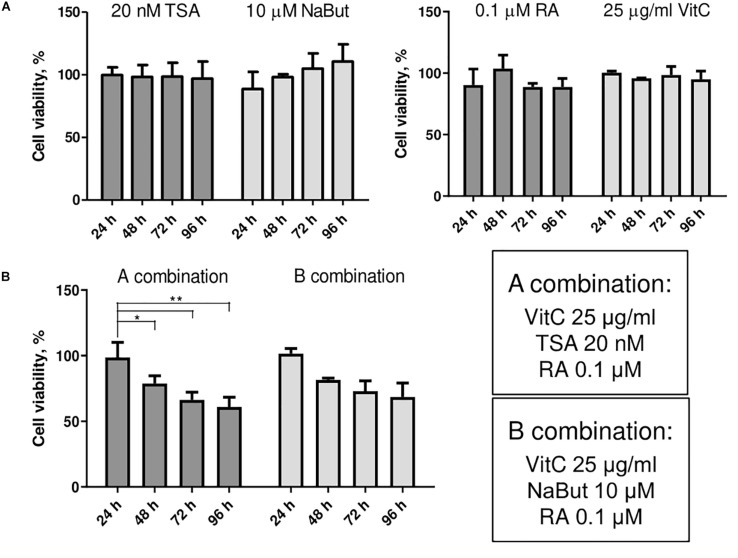
The effects of small molecules on viability of AFSCs. **(A)** Cell viability of stem cells after single small molecule treatments at 24, 48, 72, and 96 h of incubation. **(B)** Cell viability of AFSCs after treatments with small molecule combinations at 24, 48, 72, and 96 h of incubation. Cell viability was determined using MTT assay, results are presented as mean ± SD (*n* = 4), *p* ≤ 0.05 (*), *p* ≤ 0.01 (**), where not indicated: non-significant.

Small molecule treatments were tested for their toxicity as single molecules (Figure 2A) and in combinations (Figure 2B) using MTT assay. We investigated the effects of HDAC inhibitors TSA and NaBut, and multifunctional molecules RA and vitC on cell viability of AFSCs every 24 h for 4 days. The concentrations for used small molecules were chosen regarding previous studies (Huangfu et al., 2008; Esteban et al., 2010; Han et al., 2010; Hou et al., 2013). The results revealed that these compounds affect cell viability but do not induce cellular cytotoxicity at given concentrations and combinations. When treated with small molecule compounds separately cell viability did not decrease lower than 90% and treatment with NaBut even stimulated cell proliferation since cell viability improved during treatment time. After treating AFSCs with small molecule combinations a gradual decrease in cell viability was observed and after 4 days it reached around 65–75%. We also tested the effects of DNMT inhibitor decitabine (Supplementary Figure 4) as a single compound and in combinations with other small molecules. However, due to insufficient efficacy, we did not use these combinations in further studies.

**FIGURE 3:**
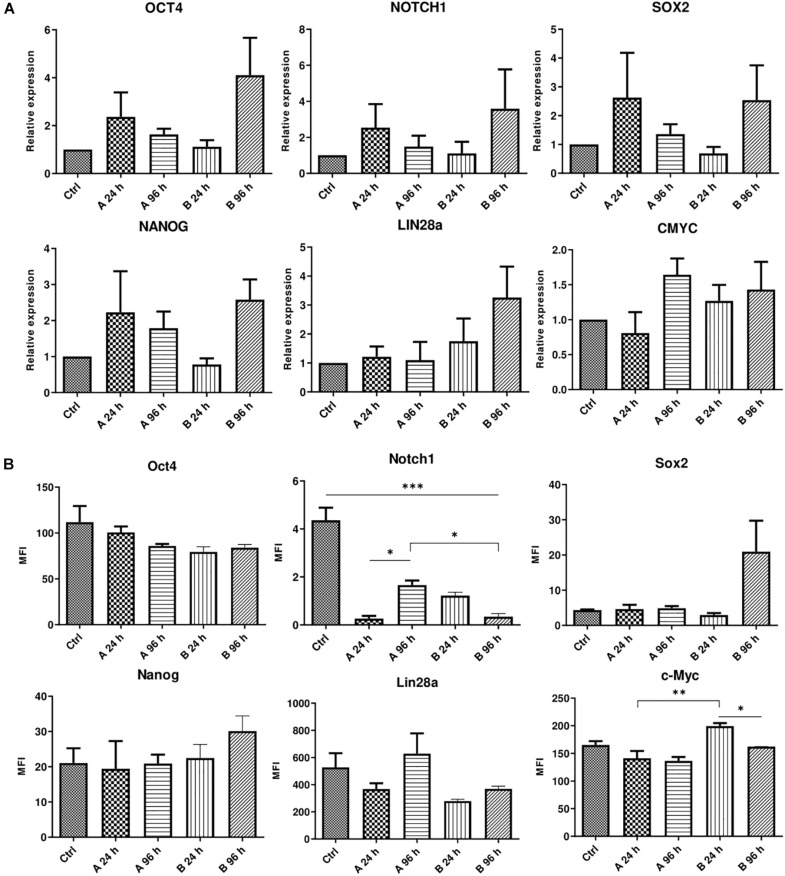
Analysis of gene and protein expression after small molecule combination treatments. **(A)** Relative gene expression of genes associated with pluripotency *OCT4*, *NOTCH1*, *LIN28a*, *SOX2*, *NANOG*, and *CMYC* after 24 and 96 h of incubation with A and B small molecule combinations. **(B)** Expression changes of corresponding proteins presented as changes of mean fluorescence intensity (MFI). Ctrl represents untreated control cells. Gene expression was determined by RT-qPCR and data, normalized to *GAPDH* are presented as fold change over untreated control. MFI was determined by flow cytometry. Results are shown as mean ± SD (*n* = 4), *p* ≤ 0.05 (*), *p* ≤ 0.01 (**), *p* ≤ 0.001 (***), where not indicated: non-significant.

AFSCs were treated with combination A (25 μg/mL vitC, 20 nM TSA, 0.1 μM RA) and B (25 μg/mL vitC, 10 μM NaBut, 0.1 μM RA) for 24 and 96 h and some variations in the expression of genes and corresponding proteins that are involved in maintaining pluripotency was observed (Figure 3). Incubation with small molecule combinations induced changes of gene expression in an adversative manner (Figure 3A). Expression levels of *OCT4*, *NOTCH1*, *SOX2*, and *NANOG* were higher with combination A after 24 h when compared to 96 h of incubation and combination B shows upregulated expression after 96 h of treatment. Expression of *LIN28a* increased only with combination B and *CMYC* showed slight upregulation with A combination after 96 h and with combination B at both time points. This indicates that even though these combinations differ by only one substance with similar function (TSA in combination A and NaBut in combination B), it can influence the cellular response and gene expression activation differently. The results of protein expression changes induced by combination A and B treatments reveal different response to small molecule stimulation. The changes in expression level of Oct4, Nanog and c-Myc are quite mild with only c-Myc displaying significant decrease with combination B after 96 h treatments when comparing to 24 h incubation. Sox2 is upregulated only with B combination after 96 h of treatments and Lin28a is downregulated except for combination A treatment after 96 h, although these expression changes are insignificant. Notch1 is significantly downregulated with both combination treatments at both time points. Differences in gene and protein expression patterns after small molecule treatments show that stimulation of gene transcription and protein translation are regulated differently.

**FIGURE 4:**
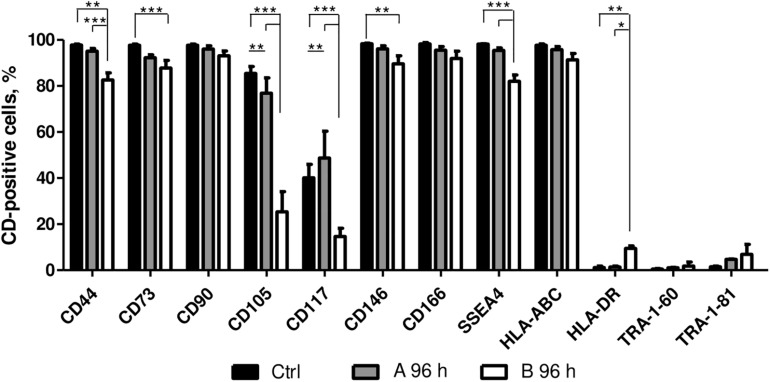
Surface marker expression analysis. Surface marker expression changes after 96 h of incubation with A and B combinations of small molecules. Ctrl represents untreated control cells. Surface marker expression was determined by flow cytometry, results are presented as mean ± SD (*n* = 3), *p* ≤ 0.05 (*), *p* ≤ 0.01 (**), *p* ≤ 0.001 (***), where not indicated: non-significant.

The effect of A and B combinations on typical mesenchymal and pluripotent stem cell surface marker and MHC class I and II surface receptor expression were tested by flow cytometry (Figure 4). After 96 h of treatment with small molecule combinations A and B the expression of CD90, CD166, HLA-ABC, TRA-1-61, and TRA-1-81 surface markers remained similar to untreated control. Compared to control cells, treatment with combination A did not have much effect on the expression of CD44, CD73, CD146, SSEA4, and HLA-DR, while with combination B the expression level of these markers decreased 10–15%, except for HLA-DR when a slight increase of approximately 10% was noted. The expression of CD105 decreased when cells were treated with both combinations and the expression of CD117 decreased with combination B, but an increase of 10% was observed with combination A.

### Changes in Metabolic Phenotype

**FIGURE 5:**
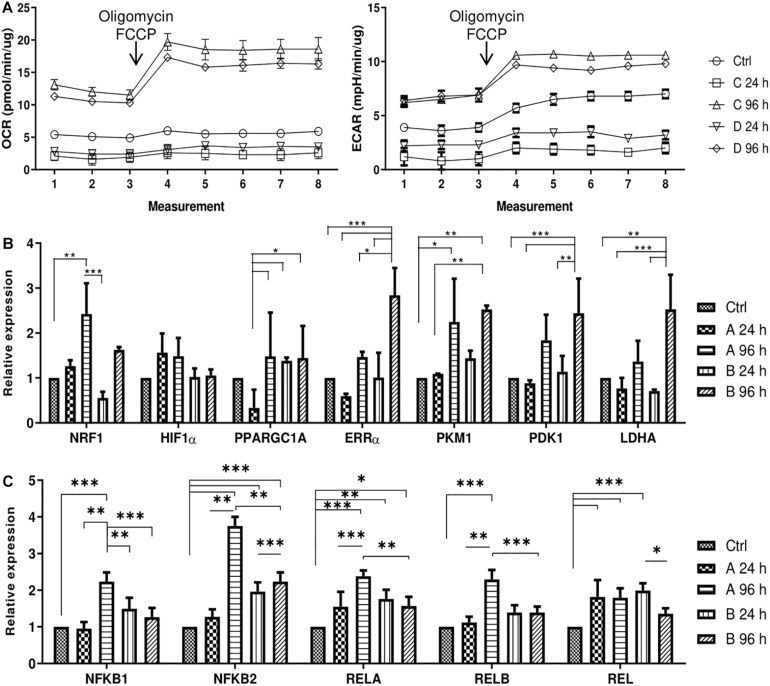
Cell energy phenotype and metabolic alterations during treatments of small molecule combinations A and B on AFSCs. **(A)** Mitochondrial respiration and glycolytic activity expressed as normalized OCR and ECAR, respectively, of control and treated cells after the addition of ETC inhibitors oligomycin and FCCP. The time in the x-axis represents time points when each measurement was taken. **(B)** The relative expression of genes, related to cell metabolism and respiration: *NRF1*, *HIF1*α, *PPARGC1A*, *ERR*α, *PKM*, *PDK1*, and *LDHA*, **(C)** the relative expression of NF-κB family genes *NFKB1, NFKB2, RELA, RELB, REL* in control and treated cells as determined by using RT-qPCR. The data were normalized to *GAPDH* and presented as a fold change over untreated control. The data in A was obtained using Seahorse XFp Extracellular Flux Analyzer (Agilent, United States) and “Cell energy phenotype test.” Results are presented as mean ± SD. *p* ≤ 0.05 (*), *p* ≤ 0.01 (**), *p* ≤ 0.001 (***), where not indicated: non-significant.

After the effects of small molecule treatments were established, the changes in metabolic profile of AFSCSs were determined. AFSCs were treated with combinations A and B for 24 and 96 h and their mitochondrial and glycolytic activity was assessed using Seahorse extracellular flux analyzer and expression of genes associated with mitochondrial respiration, glycolysis and cellular metabolism was examined (Figure 5). The rates of oxygen consumption (OCR) and extracellular acidification (ECAR) were measured simultaneously under basal and induced stressed (after addition of electron transport chain (ETC) inhibitors oligomycin and FCCP) conditions (Figure 5A). The data suggest that 96 h treatments result in more energetically active cells compared to untreated cells or 24 h incubations with small molecule combinations. Analysis of gene expression (Figure 5B) reveal that genes related to glycolysis (*ERR*α, *PKM, PDK1, LDHA*) are upregulated more significantly than genes linked to mitochondrial respiration (*NRF1*, *HIF1*α, *PPARGC1A*) and after 96 h treatments. Also, genes encoding transcription factors of NF-κB signaling pathway (*NFKB1, NFKB2, RELA, RELB, REL*) were examined (Figure 5C) and upregulated expression after treatments with both combinations was registered, especially with combination A after 96 h of treatment. The functional analysis and gene expression results suggest that small molecule combination treatments stimulate AFSCs to enter a more energetically active state.

### Assessment of Neurogenic Differentiation

**FIGURE 6:**
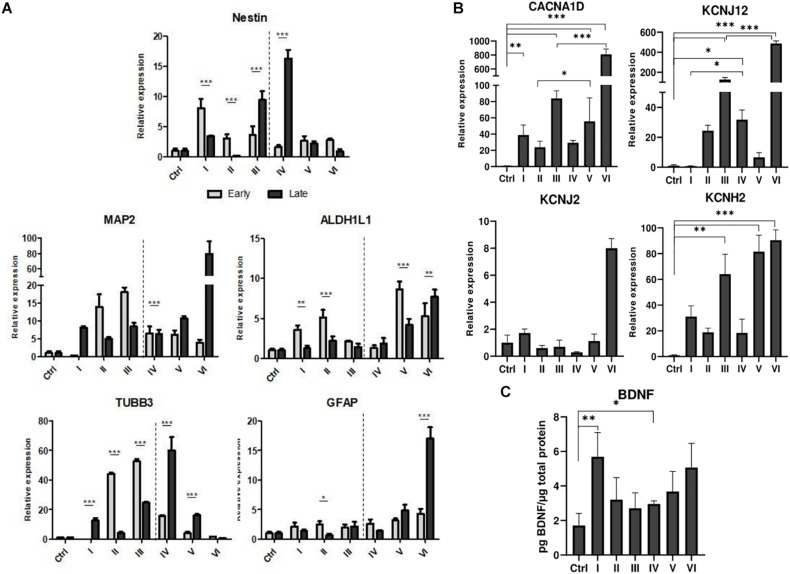
Neurogenic differentiation of AFSCs and gene expression analysis. **(A)** Relative gene expression of genes associated with neurogenic differentiation *Nestin*, *MAP2*, *TUBB3*, *ALDH1L1*, *GFAP* at early and late differentiation stages. **(B)** The expression of genes of ion channels *CACNA1D, KCNJ12, KCNJ2, KCNH2* at late differentiation stage. **(C)** Secreted BDNF levels in differentiated cells. Different differentiation medias are indicated as roman numerals I–VI (more information is listed in supporting information). Ctrl represents undifferentiated control cells. Gene expression was determined by RT-qPCR and data, normalized to *GAPDH* are presented as fold change over undifferentiated control. BDNF levels were determined by ELISA and the results were normalized to total cell protein. Results are shown as mean ± SD (*n* = 3), *p* ≤ 0.05 (*), *p* ≤ 0.01 (**), *p* ≤ 0.001 (***), where not indicated: non-significant.

**FIGURE 7:**
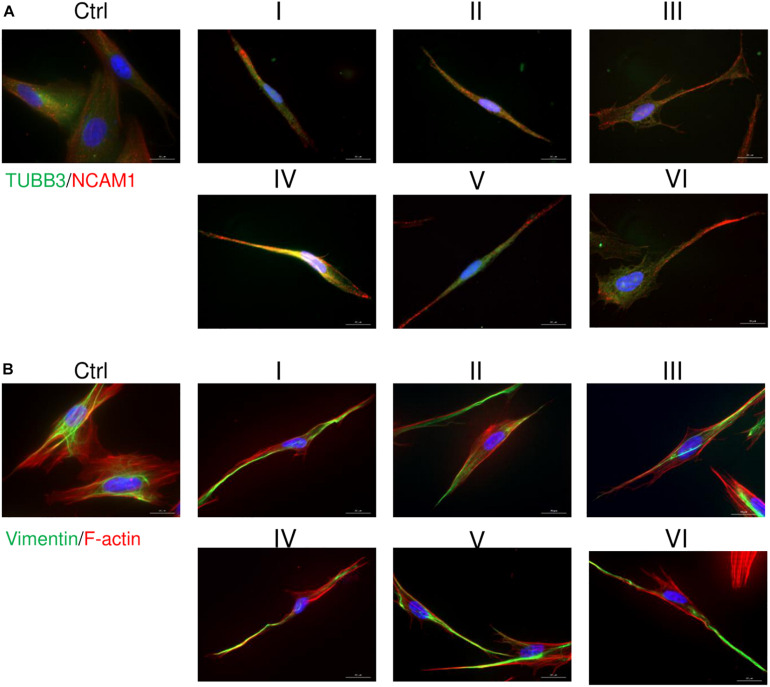
Immunofluorescence analysis of neural differentiation. Cells were differentiated using I-VI differentiation protocols and staining of TUBB3, NCAM1, Vimentin and F-actin was performed with differentiated and undifferentiated cells. **(A)** Distribution of TUBB3 (green) and NCAM1 (red). **(B)** Analysis of Vimentin (green) and F-actin (red). Nuclei are stained blue. Samples were observed using Zeiss Axio Observer fluorescence microscope, 63× magnification with immersion oil, scale bar = 20 μm.

AFSCs were induced to differentiate toward neurogenic lineage and gene expression was analyzed at early (day 7) and late (day 15) differentiation stages (Figure 6). Several differentiation medias were used containing such supplements as 1% N2 with 3 μM RA (I), 2% B27 with 3 μM RA (II) or their combination with 3 μM RA (III). Taking into account that combination C stimulates *SOX2* and *NOTCH1* expression after 24 h of treatment and other investigated genes are upregulated (except for *CMYC*) at given time point, we selected A combination and 24 h treatment for the preinduction step, especially since *SOX2* and *NOTCH1* are associated with neurogenesis. After 24 h the media with preinduction compounds was changed to differentiation medias with 1% N2 with 3 μM RA (IV), 2% B27 with 3 μM RA (V) and N2/B27 combination with 3 μM RA (VI). Morphological (Supplementary Figure 3) and gene expression changes under different conditions were observed. A preinduction step upregulated expression of astrocyte markers *ALDH1L1* and *GFAP* when compared to differentiations without preinduction step. Neural markers *MAP2* and *TUBB3* reveals varied expressional changes, when VI protocol was favorable for *MAP2* expression and IV protocol improved *TUBB3* expression (Figure 6A). Differentiation was also assessed by examining gene expression of ion channels *CACNA1D, KCNJ12, KCNJ2*, and *KCNH2* at late differentiation stage (Figure 6B). Comparing the gene expression between differentiation protocols with and without pretreatment step, the expression level of calcium ion channel *CACNA1D* was significantly upregulated when comparing protocol II and V, and protocol III and VI. Potassium ion channel *KCNJ12* also showed great upregulation when preinduction stem was added to differentiation protocol, since expression level significantly increased comparing protocols I and IV, and protocols III and VI. Secretion of BDNF was analyzed at late differentiation stage (Figure 6C) and significant increase of BDNF can be observed with protocol I and IV when comparing to undifferentiated control. Neurogenic differentiation was confirmed by staining TUBB3, NCAM1, and Vimentin (Figure 7). Differentiated cells acquire more elongated morphology which is highlighted by reorganization of TUBB3, Vimentin and F-actin, and begin to form neurite growths with NCAM1 becoming more concentrated at the cell ends. Upregulation of neural marker genes and ion channel genes suggest the beneficial effect of adding small molecule treatment step to differentiation induction protocol.

## Discussion

Ever since Takahashi and Yamanaka in 2006 discovered and developed iPSC technology (Takahashi and Yamanaka, 2006) alternative cell reprogramming approaches has been of great interest. Small molecules and their role in modifying cell fate is a rapidly developing field of study and they are an attractive alternative to viral and non-viral vectors for cellular reprogramming. One of the benefits of small molecules is their fast and mostly reversible effects that allows easy manipulation of cell treatment conditions. A vast assortment of different small molecules exists and their combination options are immeasurable.

Small molecules have been widely used for differentiation induction of stem cells from various tissues of origin. Maioli et al. (2013) achieved cardiovascular phenotype of human AFSCs by using a mixture of hyaluronic, butyric, and retinoic acids. Another study used 5-azacytidine (AZA), RA, and dimethyl sulfoxide (DMSO) to induce cardiomyogenic differentiation of fetal liver-derived MSCs (Deng et al., 2016). Small molecules are also reported to facilitate transdifferentiation. TSA and AZA was used to induce hepatic differentiation with DMSO (Cipriano et al., 2017) or to enhance differentiation (Kim et al., 2016). Also, small molecules can be used to generate more specialized stem cells, such as MScs from iPSCs or ESCs. In the study by Chen et al. (2012) used TGF-β inhibitor SB431542 to initiate mesengenic differentiation and obtain MSCs. Results of previous studies reveal the broad potential of small molecules for applications in stem cell research.

In this study, AFSCs were treaded with several selected small molecules and their combinations (Zhang et al., 2012; Qin et al., 2017; Yoshida and Yamanaka, 2017; De Angelis et al., 2018) and changes in cell and stem cell characteristics were observed. The tested combinations include HDAC inhibitors trichostatin A (TSA) and sodium butyrate (NaBut) that are shown to promote somatic cell reprogramming (Mali et al., 2010; Huang et al., 2011). It was proposed that HDAC inhibitors could replace CMYC and KLF4 factors during induction of pluripotency (Kretsovali et al., 2012). Vitamin C (vitC) was chosen based on evidence suggesting its role in DNA demethylation as a cofactor of Ten-Eleven Translocation (TET) enzymes (Esteban and Pei, 2012; Stadtfeld et al., 2012). By enhancing TET1 activity, vitC was found to indirectly promote reprogramming efficiency (Esteban et al., 2010; Blaschke et al., 2013). Retinoic acid (RA) signaling was linked to pluripotency reprogramming when enhancing effect of overexpression of RA receptor α (RARα) and γ (RARγ) was observed in iPSC derived using Yamanaka factors (Wang et al., 2011). And it has been demonstrated that the effects of RA are tightly related to used concentration. De Angelis et al. (2018) concluded that low concentrations of RA (0.5 μM) positively affected pluripotency state of hiPSCs while higher concentrations of RA (1.5 and 4.5 μM) promoted differentiation and downregulation of pluripotency markers OCT4, NANOG, and REX1. It was also demonstrated that a combination of retinoic acid and vitamin C act synergistically and boost cell reprogramming to pluripotency (Alexander et al., 2016).

Our study was focused on short-term treatments with small molecules on AFSCs. When cells were treated with small molecules individually, stable or upregulated cell viability was observed. Many papers report tendencies that agree with our results with decitabine (Pang et al., 2019), TSA (Han et al., 2013), NaBut (Panta et al., 2019), RA (Pourjafar et al., 2017), vitC (Markmee et al., 2019). Decitabine is a well-known hypomethylating agent which incorporates itself into host DNA, but is used as a drug to treat myelodisplastic syndrome and acute myeloid leukemia. And even though Öz et al. (2014) demonstrated that incorporation rates of decitabine at 100 nM are not genotoxic in myeloid leukemia cells, the effects of higher concentrations of decitabine and how its incorporation affect healthy cells are still unknown. Taking that into consideration, combinations with decitabine were excluded from further experiments.

Certain gene expression and surface marker expression are an important characteristic of stem cells. In our experiments we obtained an interesting pattern of gene expression after treatment with small molecule combinations A (vitC, TSA, RA) and B (vitC, NaBut, RA). The obtained results show that combination C leads to more upregulated expression levels of *OCT4*, *NOTCH1*, *SOX2*, and *NANOG* after 24 h compared to 96 h treatments, while combination B promote higher upregulation after 96 h when compared to 24 h treatments. Increased gene expression of *LIN28a* and was observed with combination B and *CMYC* displayed slight upregulation with combination A after 96 h and combination B at both time points. Such differences in gene expression upregulation could be linked with different HDAC inhibitors present in used combinations of small molecules. Differential effects of TSA and NaBut were reported in breast cancer cells (Kalle and Wang, 2019), but more information on how these molecules influence pluripotency associated gene activation in lacking. At protein level investigated markers show different response to small molecule treatments. Oct4, Nanog, and c-Myc show minor changes in the expression levels with significant decrease of c-Myc with combination B comparing 96–24 h treatment and significant downregulation of Notch1 is induced by small molecule treatments.

A set of surface markers related with multipotent mesenchymal stem cells were examined. The most profound effect was noted after 96 h incubation with B combination when significant decrease of CD positive cells were observed for such markers as CD44, CD73, CD105, CD117, CD146, SSEA4. Na et al. (2015) report that CD105 expression is regulated by Notch signaling pathway. They demonstrate that inhibition of Notch signaling leads to reduction of CD105 expression, in contrast, our results show *NOTCH1* upregulation after 96 h with B combination, thus meaning that other mechanisms are in play of regulating the expression of this surface marker. One more factor that can influence expression of CD105 is the concentration of serum in culture media. A study by Mark et al. (2013) demonstrated that only 50% of bone marrow stem cells cultured under serum-free conditions were positive for CD105, when nearly 100% of cells cultured in media containing serum were positive for CD105.

Cellular metabolism and metabolites are closely involved in regulating epigenetic state of the cell and epigenetics have a crucial role in regulating metabolic profile of the cell. Also, it has been established that during somatic cell reprogramming upregulation of genes associated with glycolytic metabolism occurs even before the expression of pluripotency genes (Folmes et al., 2011; Cao et al., 2015). In our experiment treatment with small molecule combinations result in more energetically active cells as evident by increased oxygen consumption (OCR) and extracellular acidification (ECAR) rates after 4 days. Also, both oxidative phosphorylation (*NRF1*, *HIF1*α, *PPARGC1A*) and glycolytic (*ERR*α, *PKM*, *PDK1*, *LDHA*) genes are upregulated after 96 h treatments. Small molecules are known to improve stem cell function by regulation of cellular metabolism (Son et al., 2018). And several studies suggest that the initiation phase of cell reprogramming could be characterized by a phenomenon called transient hyper-energetic metabolism, which is a hybrid of high OxPhos and high glycolysis. Cacchiarelli et al. (2015) report metabolism-related genes show peak levels of expression at an early stage of reprogramming. Also, Kida et al. (2015) have linked ERRα and ERRγ expression to iPSC generation. Their study identified transient upregulation of ERRα and ERRγ, which are typically expressed in oxidative tissues, in the early stages of reprogramming and showed that the transient OxPhos burst and increased glycolysis are essential for reprogramming (Kida et al., 2015). Additionally, we examined gene expression of NF-κB family, since it has been associated with cellular metabolism. It was shown that depletion of IKKa or RelB, which are important components of NF-κB signaling pathway, resulted in reduced mitochondrial content and function (Bakkar et al., 2012). Also, it was determined that p65 or RelA, another protein of NF-κB family, promotes the mitochondrial expression of cytochrome c oxidase 2 assembly factor and oxidative phosphorylation (Mauro et al., 2012). It was also proposed that glycolysis stimulates IKK/NF-κB activity, as revealed by reduced IKK activity in the presence of a glycolytic inhibitor and increased IKK activity after GLUT3 expression (Kawauchi et al., 2008). Increased levels of OCR and ECAR as well as upregulated expression of genes associated with cellular metabolism would suggest that small molecule treatments increase energetic needs of AFSCs, which could be similar to those linked to early reprogramming stages.

Because of an increase in *SOX2* and *NOTCH1* expression, A combination was chosen for a preinduction step in neurogenic differentiation induction. SOX2 is a transcription factor known for its role in neuroectoderm development (Sarlak and Vincent, 2016) and NOTCH1 is a receptor and its signaling is crucial in neurogenesis (Lathia et al., 2008), thus the upregulation of these genes could potentially promote neurogenic differentiation of AFSCs. To test this theory, neurogenic differentiation was induced by using two commercially available supplements N2 and B27 and with the addition of RA (Janesick et al., 2015), without a preinduction step and with a 24 h preinduction, then expression of neurogenic differentiation associated genes was analyzed. In our experiment small molecule pretreatment improved the expression of astrocytic genes *ALDH1L1* and *GFAP*, neural markers *MAP2* benefited from preinduction step when induced with N2/B27 supplements and *TUBB3* showed increased expression after small molecule treatment when induced using N2 supplement. Also, the preinduction step proved to benefit the expression levels of ion channel genes. Small molecule treatment boosted expression of calcium ion channel *CACNA1D* when differentiation was induced with B27 and N2/B27 supplements. While potassium ion channel gene *KCNJ12* showed an increase in expression when N2 and N2/B27 supplements were used for neurogenic differentiation. Neurogenic differentiation was confirmed by significantly increased levels of secreted BDNF when using N2 supplement alone or with the preinduction step. Also, morphological changes were visualized by staining TUBB3, NCAM1, Vimentin and F-actin. Differentiated AFSCs become more elongated as evident by reorganization of structural cytoskeleton proteins TUBB3, Vimentin and F-actin, and possible neurites begin to form as NCAM1 is becoming more concentrated at the cell ends. Using small molecules to improve neurogenic differentiation by targeting signaling pathways (Song et al., 2018) or epigenetic regulation (Xu et al., 2019) has been reported, but evidence of similar effect when using the same small molecules as used in this study is scarce.

## Conclusion

To conclude, small molecules are an important tool in cell biology, cancer research, they are investigated as potential drugs for many disorders. Also, small molecules can be used to enhance cellular properties of stem cells. Our investigated small molecule combinations upregulated genes related to pluripotency, treatments lead to more energetically active cells, and pretreatment step deemed beneficial for neurogenic differentiation. A vast selection of small molecules exists and many different combinations could lead to different effects. Many studies are focused on establishing these effects and bringing small molecules closer to clinical use.

## Data Availability Statement

The raw data supporting the conclusions of this article will be made available by the authors, without undue reservation.

## Ethics Statement

The studies involving human participants were reviewed and approved by the Ethics Committee of Biomedical Research of Vilnius District. The patients/participants provided their written informed consent to participate in this study.

## Author Contributions

AZ, VB, and RN: conception and design, collection of data, analysis, and interpretation, and writing original draft of manuscript. DŽ and IJ: data collection and interpretation. All authors contributed to the article and approved the submitted version.

## Conflict of Interest

The authors declare that the research was conducted in the absence of any commercial or financial relationships that could be construed as a potential conflict of interest.
