# Supplementary material for: Small Molecule Treatments Improve Differentiation Potential of Human Amniotic Fluid Stem Cells
Source: Front Bioeng Biotechnol. 2021 Feb 22;9:623886. doi: 10.3389/fbioe.2021.623886 (PMC7937811; doi:10.3389/fbioe.2021.623886)
Supplement: Supplementary file 1 [file Table_1.DOCX]

Supplementary Material


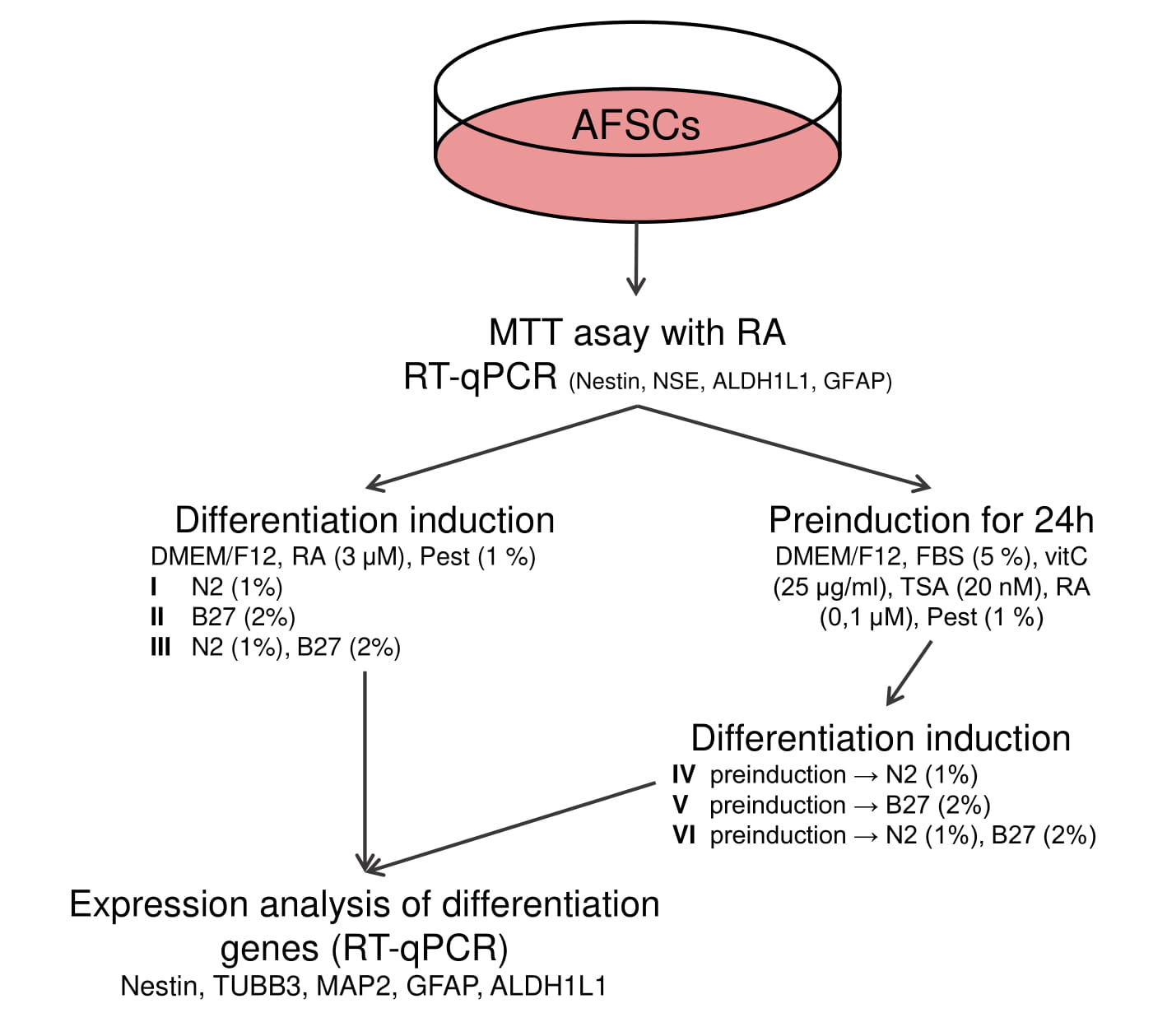


**Supplementary Figure 1**. Schematics of experiment design for neurogenic differentiation of AFSCs. Optimal RA concentration was determined by MTT assay and expressional changes of neurogenic genes. AFSCs were induced to differentiate using commercial supplements and RA or treated with a preinduction step consisting of incubation with a combination of small molecules (25 µg/ml vitC, 20 nM TSA and 0.1 µM RA) for 24 hours followed by differentiation induction with commercial supplements and RA. During differentiation expression levels of Nestin, TUBB3, MAP2, GFAP and ALDH1L1 were evaluated.


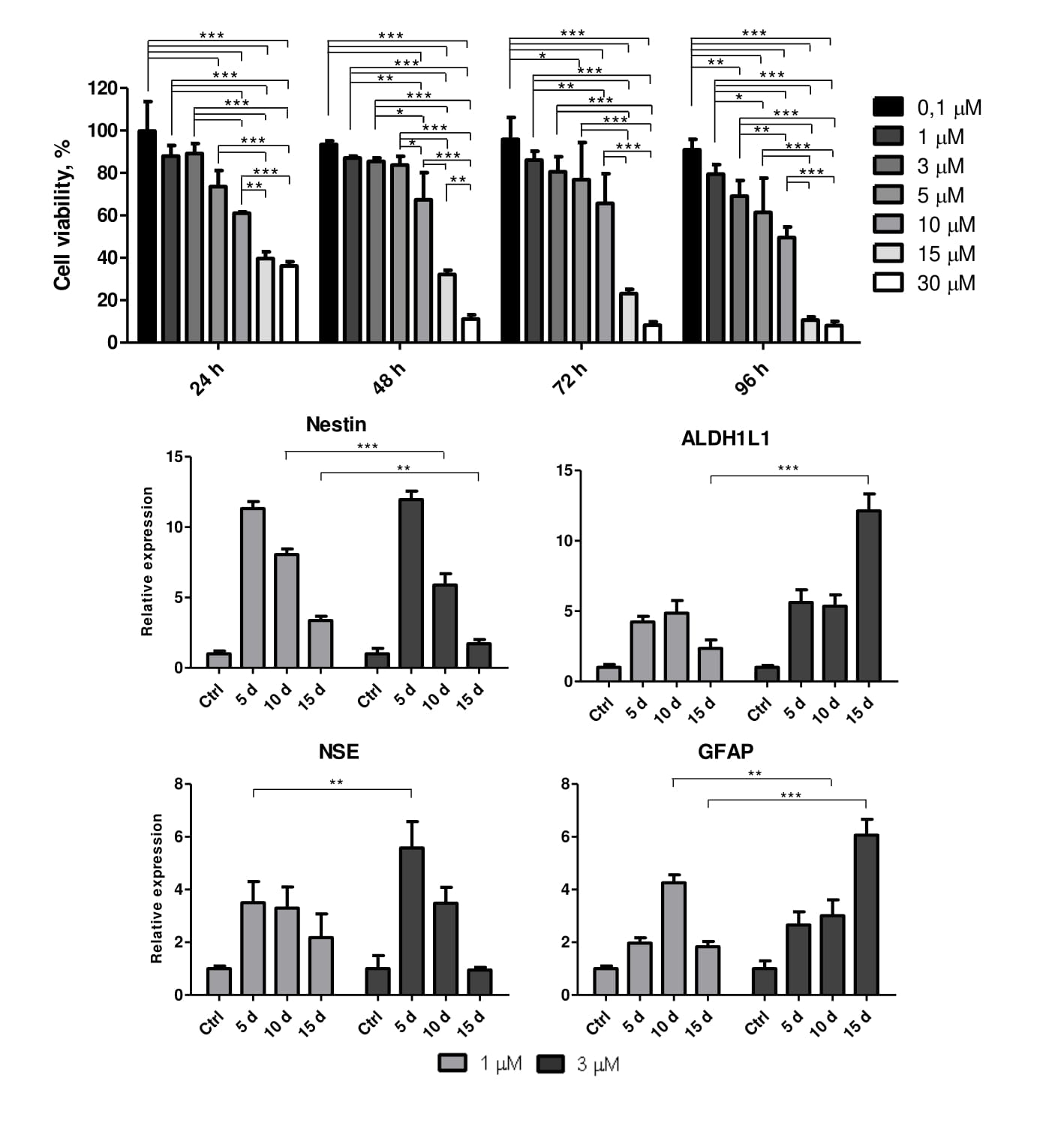


**Supplementary Figure 2.** Optimization of neurogenic differentiation conditions. The effects of different concentrations of RA (0,1 µM to 30 µM) on cell viability were tested using MTT assay. 1 and 3 µM concentrations of RA were selected for evaluation of expression levels of Nestin, ALDH1L1, NSE and GFAP genes. Ctrl represents untreated control cells. Gene expression was determined by RT-qPCR and data, normalized to GAPDH are presented as n‐fold change over control. Results are shown as mean ± SD (n = 3), p≤0.05 (*), p≤0.01 (**), p≤0.001 (***), where not indicated: non-significant.


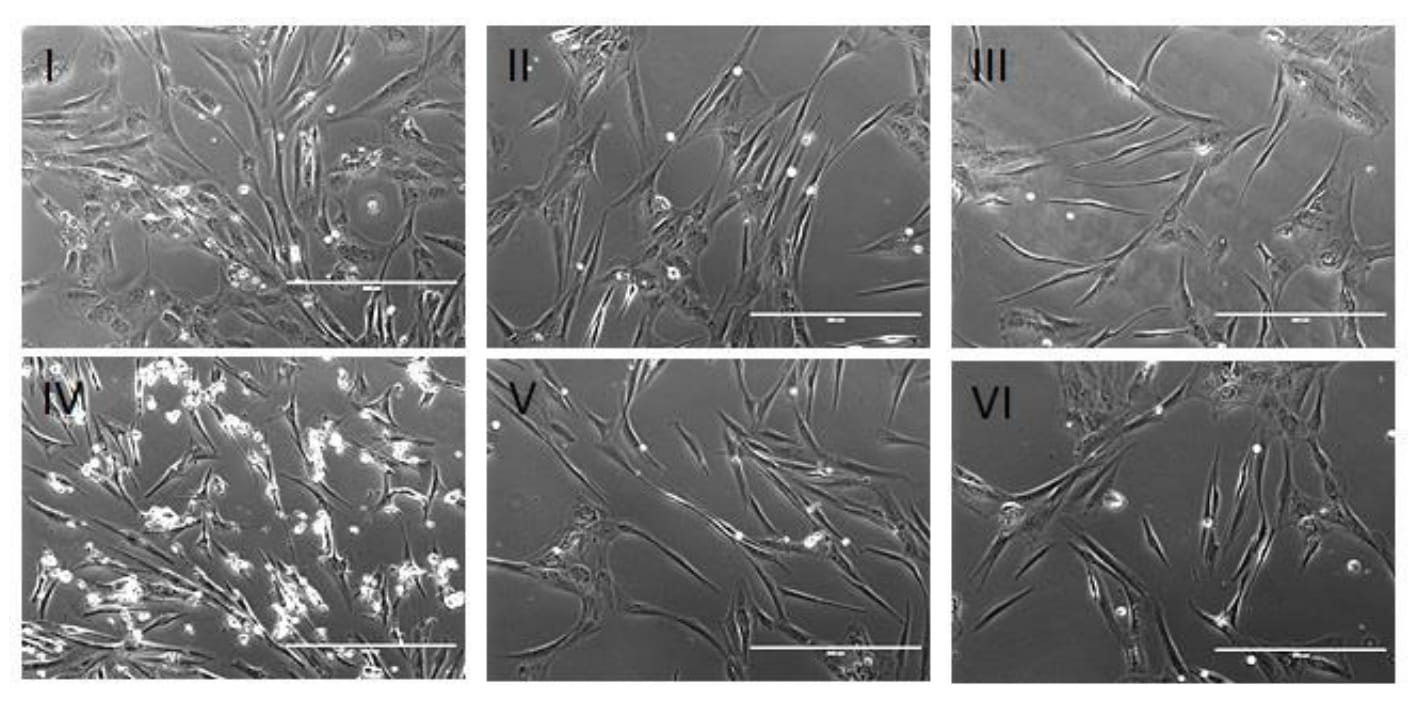


**Supplementary Figure 3.** Morphology of AFSCs differentiated towards neurogenic lineage. Differentiation medias consisted of 1 % N2 with 3 µM RA (I), 2 % B27 with 3 µM RA (II) or their combination with 3 µM RA (III). AFSCs were treated with combination C (25 µg/mL vitC, 20 nM TSA, 0,1 µM RA) for 24 hours and then differentiated with medias supplemented with 1 % N2 with 3 µM RA (IV), 2 % B27 with 3 µM RA (V) and N2/B27 combination with 3 µM RA (VI) for 15 days. Scale bar 400 µm.


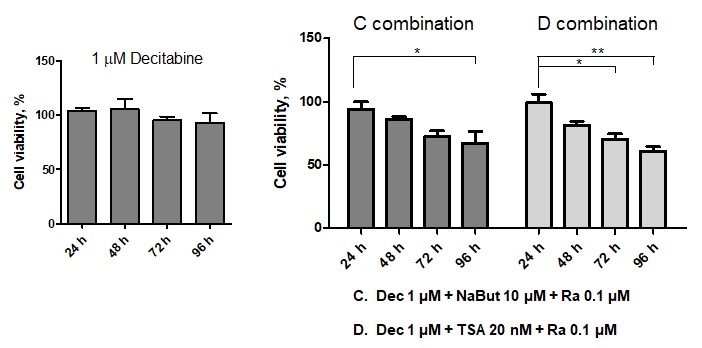


**Supplementary Figure** **4**. The effects of small molecule decitabine on viability of AFSCs. Cell viability of stem cells after decitabine treatments separately or in combinations at 24, 48, 72 and 96 hours of incubation. Cell viability was determined using MTT assay, results are presented as mean ± SD (n = 4), p≤0.05 (*), p≤0.01 (**), where not indicated: non-significant.

**Supplementary table 1**. Sequences of used primers.

| No. | Name | Primer sequence |
| --- | --- | --- |
| 1. | GAPDH | F 5‘-AGTCCCTGCCACACTCAG  R 5‘-TACTTTATTGATGGTACATGACAAGG |
| 2. | SOX2 | F 5‘-TGGACAGTTACGCGCACAT  R 5‘-CGAGTAGGACATGCTGTAGGT |
| 3. | OCT4 | F 5‘-ACATGTGTAAGCTGCGGC  R 5‘-GTTGTGCATAGTCGCTGCTTG |
| 4. | NANOG | F 5‘-AGATGCCTCACACGGAGACT  R 5‘-GTTTGCCTTTGGGACTGGTG |
| 5. | KLF4 | F 5‘-CACATTAATGAGGCAGCCACC  R 5‘-AAGTCGCTTCATGTGGGAGAG |
| 6. | NOTCH1 | F 5‘-GGTGAGACCTGCCTGAATG  R 5‘-GTTGGGGTCCTGGCATC |
| 7. | LIN28A | F 5‘-TTGTCTTCTACCCTGCCCTCT  R 5‘-GAACAAGGGATGGAGGGTTTT |
| 8. | MYC | F 5‘-AATGAAAAGGCCCCCAAGGTAGTTATCC  R 5‘-GTCGTTTCCGCAACAAGTCCTCTTC |
| 9. | NRF1 | F 5‘-AGGCTGGGGGAAAGAAAG  R 5‘-CCAACCTGGATAAGTGAGAC |
| 10. | HIF1Α | F 5‘-CCAACAGTAACCAACCTCAG  R 5‘-TCCTGTGGTGACTTGTCCTT |
| 11. | PPARG1CA | F 5‘-GCAATTGAAGAGCGCCGTGTGA  R 5‘-CTGTCTCCATCATCCCGCAGAT |
| 12. | ERRΑ | F 5‘-AGGGTTCCTCGGAGACAGAG  R 5‘-TCACAGGATGCCACACCATAG |
| 13. | PKM | F 5‘-ATGTCGAAGCCCCATAGTGAA  R 5‘-TGGGTGGTGAATCAATGTCCA |
| 14. | PDK1 | F 5‘-GAGAGCCACTATGGAACACCA  R 5‘-GGAGGTCTCAACACGAGGT |
| 15. | LDHA | F 5‘-ATGGCAACTCTAAAGGATCAGC  R 5‘-CCAACCCCAACAACTGTAATCT |
| 16. | NFKB1 | F 5’-GAAGCACGAATGACAGAGGC  R 5’-GCTTGGCGGATTAGCTCTTTT |
| 17. | NFKB2 | F 5’-ATGGAGAGTTGCTACAACCCA  R 5’-CTGTTCCACGATCACCAGGTA |
| 18. | RELA | F 5’-ATGTGGAGATCATTGAGCAGC  R 5’-CCTGGTCCTGTGTAGCCATT |
| 19. | RELB | F 5’-CCATTGAGCGGAAGATTCAACT  R 5’-CTGCTGGTCCCGATATGAGG |
| 20. | REL | F 5’-AAAGACTGCAGAGACGGCTA  R 5’-CTCACCACATTGAGGTCACA |
| 21. | NESTIN | F 5‘-CTGCTACCCTTGAGACACCTG  R 5‘-GGGCTCTGATCTCTGCATCTAC |
| No. | Name | Primer sequence |
| 22. | MAP2 | F 5‘-CCAATGGATTCCCATACAGG  R 5’-TCCTTGCAGACACCTCCTCT |
| 23. | TUBB3 | F 5‘-CTCAGGGGCCTTTGGACATC  R 5’-CAGGCAGTCGCAGTTTTCAC |
| 24. | ALDH1L1 | F 5‘-GCCTGGCTTCTGGTGTCTTC  R 5‘-GCCACGTCGGTCTTGTTGTA |
| 25. | GFAP | F 5‘-GGCAAAAGCACCAAAGACGG  R 5‘-GGCGGCGTTCCATTTACAAT |
| 26. | CACNA1D | F 5‘-GGGCAATGGGACCTCATAAATAA  R 5‘-TTACCTGGTTGCGAGTGCATTA |
| 27. | KCNJ12 | F 5‘-GCCAGCTAGGCTCTGTTTGTG  R 5‘-CTGAGACACATCTCTAAGGTAC |
| 28. | KCNJ2 | F 5‘-TGTTGGGTTTGACAGTGGAA  R 5‘-CCCACAGGATTTCATTTGCT |
| 29. | KCNH2 | F 5‘-CATTGGCTCCCTCATGTATGCT  R 5‘-GCGTGCTGGAAGTACTCCTCG |
